# Heart toxicity from breast cancer radiotherapy: Current findings, assessment, and prevention

**DOI:** 10.1007/s00066-018-1378-z

**Published:** 2018-10-11

**Authors:** Marc D. Piroth, René Baumann, Wilfried Budach, Jürgen Dunst, Petra Feyer, Rainer Fietkau, Wulf Haase, Wolfgang Harms, Thomas Hehr, David Krug, Arnd Röser, Felix Sedlmayer, Rainer Souchon, Frederik Wenz, Rolf Sauer

**Affiliations:** 1 Helios University Hospital Wuppertal, Witten/Herdecke University, Heusnerstraße 40, 42283 Wuppertal, Germany; 2 St. Marien-Krankenhaus Siegen, Siegen, Germany; 3 Heinrich-Heine-University Hospital Düsseldorf, Düsseldorf, Germany; 4 University Hospital Schleswig-Holstein, Kiel, Germany; 5 Vivantes Hospital Neukoelln, Berlin, Germany; 6 University Hospital Erlangen, Erlangen, Germany; 7 formerly St.-Vincentius-Hospital Karlsruhe, Karlsruhe, Germany; 8 St. Claraspital Basel, Basel, Switzerland; 9 Marienhospital Stuttgart, Stuttgart, Germany; 10 University Hospital Heidelberg, Heidelberg, Germany; 11 Paracelsus Medical University Hospital Salzburg, Salzburg, Austria; 12 formerly University Hospital, Tübingen, Germany; 13 University Hospital Mannheim, Medical Faculty, Heidelberg University, Mannheim, Germany; 14 Heidelberg Institute for Radiation Oncology (HIRO), National Center for Radiation Research in Oncology (NCRO), Heidelberg, Germany

**Keywords:** Heart toxicity, Breast cancer radiotherapy, Breast cancer, Mean heart dose, LAD, Herztoxizität, Bestrahlung bei Brustkrebs, Brustkrebs, Mittlere Herzdosis, LAD

## Abstract

**Background:**

Late cardiac toxicities caused by (particularly left-sided) breast radiotherapy (RT) are now recognized as rare but relevant sequelae, which has prompted research on risk structure identification and definition of threshold doses to heart subvolumes. The aim of the present review was to critically discuss the clinical evidence on late cardiac reactions based on dose-dependent outcome reports for mean heart doses as well as doses to cardiac substructures.

**Methods:**

A literature review was performed to examine clinical evidence on radiation-induced heart toxicities. Mean heart doses and doses to cardiac substructures were focused upon based on dose-dependent outcome reports. Furthermore, an overview of radiation techniques for heart protection is given and non-radiotherapeutic aspects of cardiotoxicity in the multimodal setting of breast cancer treatment are discussed.

**Results:**

Based on available findings, the DEGRO breast cancer expert panel recommends the following constraints: mean heart dose <2.5 Gy; D_mean_LV (mean dose left ventricle) < 3 Gy; V5_LV_ (volume of LV receiving ≥5 Gy) < 17%; V23_LV_ (volume of LV receiving ≥23 Gy) < 5%; D_mean_LAD (mean dose left descending artery) < 10 Gy; V30_LAD_ (volume of LAD receiving ≥30 Gy) < 2%; V40_LAD_ (volume of LAD receiving ≥40 Gy) < 1%.

**Conclusion:**

In addition to mean heart dose, breast cancer RT treatment planning should also include constraints for cardiac subvolumes such as LV and LAD. The given constraints serve as a clinicians’ aid for ensuring adequate heart protection. The individual decision between sufficient protection of cardiac structures versus optimal target volume coverage remains in the physician’s hand. The risk of breast cancer-specific mortality and a patient’s cardiac risk factors must be individually weighed up against the risk of radiation-induced cardiotoxicity.

## Abstract (de)

**Hintergrund:**

Kardiale Spättoxizitäten aufgrund einer Bestrahlung der Brust (insbesondere linksseitig) sind als seltene aber relevante Folgeerscheinungen darstellbar, was weitere Untersuchungen mit Identifikation aussagekräftiger Risikostrukturen sowie folgend die Definition von Grenzdosen kardialer Subvolumina sinnvoll erscheinen lässt.

**Methoden:**

Ein Literaturreview wurde durchgeführt, um die klinische Evidenz der strahlentherapieinduzierten Herztoxizität zu beleuchten. Die mittlere Herzdosis sowie auch kardiale Substrukturen wurden fokussiert, basierend auf Berichten mit Dosis-Wirkungs-Abhängigkeiten. Des Weiteren wird ein Überblick der technischen Möglichkeiten der Herzschonung gegeben und nichtradiotherapeutische Aspekte der Kardiotoxizität in der multimodalen Behandlung des Mammakarzinoms werden kommentiert.

**Ergebnisse:**

Basierend auf den verfügbaren Daten empfiehlt das Expertenpanel Mamma der DEGRO folgende Grenzwerte, um das Herz so effektiv wie möglich zu schützen: mittlere Herzdosis <2,5 Gy; D_mean_ LV (mittlere Dosis linksventrikulär) < 3 Gy; V5_LV_ (Volumen des linken Ventrikels, das ≥5 Gy erhält) < 17%; V23_LV_ (Volumen des linken Ventrikels, das ≥23 Gy erhält) < 5%; D_mean_ LAD (mittlere Dosis der linken anterioren absteigenden Koronararterie) < 10 Gy; V30_LAD_ (Volumen der LAD, das ≥30 Gy erhält) < 2%; V40_LAD_ (Volumen der LAD, das ≥40 Gy erhält) < 1%.

**Schlussfolgerung:**

Zusätzlich zur mittleren Herzdosis sollten kardiale Subvolumina, wie linker Ventrikel und LAD, mit entsprechenden Grenzwerten in die Bestrahlungsplanung des Mammakarzinoms einbezogen werden. Die Dosisgrenzwerte sollen dem Kliniker helfen, das Herz bei der Bestrahlungsplanung der Brust adäquat zu schützen. Die individuelle Entscheidung zwischen einer suffizienten Schonung kardialer Strukturen einerseits und der optimalen Zielvolumenabdeckung andererseits bleibt in der Hand des Arztes. Das Risiko der brustkrebsspezifischen Mortalität und die sonstigen kardialen Risikofaktoren des Patienten müssen individuell gegenüber möglichen strahleninduzierten Herztoxizitäten abgewogen werden.

## Background

After breast-conserving surgery (BCS), whole-breast irradiation (WBI) with a total dose of 50 Gy reduces the local recurrence rate by 70–88% [1, 2]. Moreover, a 5.3% reduction in overall mortality after 15 years could be shown in favor of adjuvant radiotherapy (RT) [3].

However, it is suspected that RT of left-sided breast cancer might lead to relevant cardiac toxicities [3–5]. In early trials including breast RT, an increase in the number of cardiac deaths was observed [4] and cardiac mortality was higher in left-sided breast cancer patients than in right-sided disease [5–7]. These trials predominantly used older RT techniques, resulting in considerable doses to heart subvolumes [6–9].

Major advances in RT techniques throughout the past decades, such as three-dimensional (3D) treatment planning, have led to a continuous reduction in radiation dose to the heart. Taylor et al. comparatively analyzed mean heart doses from left tangential RT to cardiac structures over several decades, and described reductions in mean heart dose from 13.3 Gy in the 1970s, to 4.7 Gy in the 1990s, and 2.3 Gy in 2006 [10–12]. This decrease seems to have resulted in a very low risk of death caused by radiation-induced heart disease (RIHD), at least for women without cardiac risk factors [13].

**Fig. 1:**
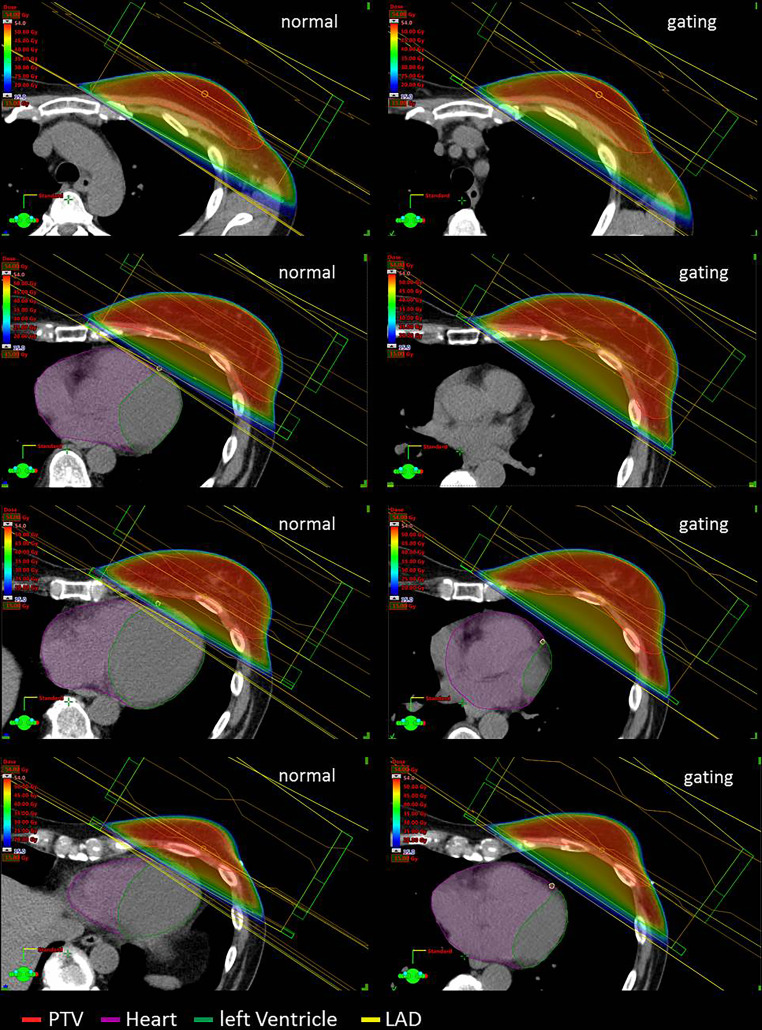
Three-dimensional (3D) treatment plans with and without gating (transverse slides, dose-wash). Left side: 3D treatment planning without deep-inspiration breathold (DIBH) with normal breathing; right side: the same patient planned using gated breathing with DIBH. Planning target volume (*PTV*) contoured in *red*, heart contoured in *purple*, left ventricle contoured in* green*, left anterior descending artery (*LAD*) contoured in *yellow*

**Fig. 2:**
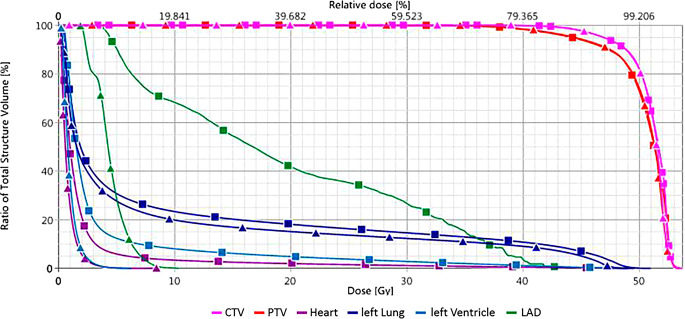
Dose–volume histogram from the two treatment plans shown in Fig. 1. Graphs with *triangles*: with deep inspiration breathold (DIBH); graphs with *squares*: without DIBH. Planning target volume (*PTV*) in *red*, clinical target volume (*CTV*) in *pink*, whole heart in *purple*, left ventricle in *light blue*, left anterior descending artery (*LAD*) in *green*, left lung in *dark blue*

However, it remains to be considered that despite low mean heart doses, relevant areas of the heart can be exposed to doses between 40 and 50 Gy [14], as shown exemplarily in Figs. 1 and 2. Mean heart dose—the only parameter reported in earlier studies—does not seem to reliably reflect the cardiac risk in many cases [15]. Nevertheless, the results of a recently performed practice pattern survey showed that most of the participating radiotherapists consider the mean heart dose to be the most important dose parameter related to heart sparing in breast cancer RT [16].

The dose values from the treatment planning shown in Fig. 1 and 2 ist showed in detail in Table 1. The aim of the present paper is to critically discuss whether mean heart dose should continue to be regarded as the most relevant parameter for prediction of cardiac toxicities or if dose constraints for substructures of the heart are more relevant. Furthermore, we want to give an overview of techniques to protect the heart and comment on non-radiotherapeutic aspects of cardiotoxicity in the multimodal setting of breast cancer treatment.

**Table 1:** Comparison of mean and maximum doses to target volumes, heart and heart substructures, and left lung achieved performing treatment planning without and with DIBH (Fig. 1)

	Mean dose (Gy)		Maximum dose (Gy)	
Structure	*no DIBH*	*DIBH*	*no DIBH*	*DIBH*
PTV	50.4	50.4	52.8 (D_2%_)	52.9 (D_2%_)
CTV	51.0	50.9	52.9 (D_2%_)	52.9 (D_2%_)
Whole heart	2.1	0.8	49.1	8.6
Left ventricle	3.8	1.0	49.1	6.2
LAD	19.3	4.4	44.6	10.4
Left lung	9.5	8.2	51.0	49.6

*DIBH* deep inspiration breathold; *LAD* left anterior descending artery; *D*_*2%*_ dose exceeding ≤ 2% of the volume; *PTV* planning target volume; *CTV* clinical target volume

## Methods

### Assessment of cardiac toxicities

A literature review was performed to examine the published clinical evidence on radiation-induced heart toxicities (summarized in Table 2).

**Table 2:** Summary of publications focusing radiation-induced heart toxicity based on several findings, and deduced doses for heart and subvolumes

	Year of treatment	Method of detection	Time to effects	Heart or subvolume dose	Effect
Darby et al. 2013 [29]	1958–2001	Retrospective population-based case–control study	Within 20 years/within first 4 years post RT	Per 1 Gy mean heart dose Note: no significances for mean heart dose < 2 Gy	Increase of relative risk for mayor coronary events: 7.4%/16.3%
van den Bogaard et al. 2017 [30]	2005–2008	–	Within 9 years post RT	Per 1 Gy mean heart dose	16.5% increase in cumulative incidence (HR 1.165) for acute coronary events
van den Bogaard et al. 2017 [30]	2005–2008	–	Within 9 years post RT	V5_LV_: 29.3% vs. 16.9%	Acute coronary event vs. no
Carr et al. 2005 [32]	1937–1965	Retrospective analysis, estimating cardiac data	22.5 years	Whole heart dose ≥ 2.8 (2.6–3) Gy and 5% volume of the heart (apex) ≥12.9 (12–13.9) Gy	Significant increase in coronary heart disease: relative risk 1.54; 95% CI: 1.15–2.06
Marks et al. 2005 [24]	1998–2001	Cardiac SPECT imaging	6–24 months	Cardiac apex included into the radiation fields (i. e., >23–25 Gy; 1.8–2 Gy per day)	27–42% new perfusion defects in cardiac apex
				<5% vs. ≥5% of the left LV into the radiation fields	Perfusion defects in 10–20% vs. 50–60% of patients
Nilsson et al. 2012 [22]	1970–2003	Angiography	10.3 years	Coronary arteries within (or near) the tangential radiation field, so called hotspot areas: mid, distal, and distal diagonal branch of LAD	Stenosis in LAD (mid, distal and distal diagonal branch of LAD)
Moignier et al. 2015 [23]	2000–2008	Coronary CT angiography	Median 6 years	Coronary artery segments: median 30.3 Gy vs. 26.3 Gy	Coronary stenosis
Skyttä et al. 2015 [28]	2011–2013	Serum troponin T	9 months (mean)	Mean heart dose: 4 Gy vs. 2.8 Gy	Increase of serum troponin T (hscTNT) > 30%
				Mean LV dose: 6.7 vs. 4.5 Gy	
				Mean LAD dose: 23.8 vs. 17.5 Gy	
				V20_LAD_: 55.4% vs. 36.2%	
				V30_LAD_: 45% vs. 29.3%	
Erven et al. 2011 [25]	–	Regional strain value, detected by Doppler echocardiography	Immediately after RT and 2 months after RT	Left apical ventricular segments >3 Gy vs. <3 Gy	Significant decrease in strain respectively systolic myocardial function

*LV* left ventricle; *LAD* left anterior descending artery; *Vx*_*LV*_ percent of left ventricle volume receiving ≥ x Gy, *HR* hazard ratio, *SPECT* single-photon emission computed tomography, *hscTNT* high-sensitivity cardiac troponin T, *RT* radiotherapy

## Results

### Pathophysiological findings

Even though the pathophysiological mechanisms of radiation-induced heart damage are incompletely understood, it is known that multiple effects contribute to heart toxicity. In vitro and in vivo studies show radiogenic effects on the micro- and macrovascular systems. These effects include inflammation, oxidative effects, cytokine activity, and endothelial damage, and lead to an accelerated atherosclerotic process [17]. The pathophysiological scenario of radiation-induced cardiovascular disease encompasses direct damage to the coronary arteries, fibrosis of the pericardium and myocardium, microvascular damage, and valve stenosis [18–20]. In this context, atherosclerotic changes play a major role. Endothelial cells are sensitive to radiation and radiation doses ≥ 2 Gy can induce inflammatory effects which result in arteriosclerosis [20, 21]. This coronary damage can lead to perfusion deficiencies, ischemia, and myocardial fibrosis [20]. Beyond this, no valid evidence exists for radiation-induced atherosclerotic effects [20].

### Angiographic findings

To assess a possible correlation between breast RT and the subsequent locations of coronary stenoses, Nilsson et al. [22] investigated 199 women with invasive breast cancer or ductal carcinoma in situ within a cohort irradiated between 1970 and 2003, who then received coronary angiography during the period from 1990 to 2004. The median interval from breast cancer to coronary angiography was 10.3 years (25^th^ percentile 5.4 years; 75% percentile 15.8 years). During the study period (1970 to 2003), several different RT regimens were used. Therefore, Nilsson et al. divided the respective RT concepts into high or low risk, depending on the estimated doses to so called hotspot areas, which were defined as follows: the proximal right coronary artery (prox. RCA) and the “mid and distal left anterior descending artery and distal diagonal” (mdLAD + dD). The authors found an increase in clinically significant coronary artery stenoses in the predefined hotspot areas in patients who underwent left-sided WBI/chest wall RT compared to patients who did not receive RT to these areas. Radiation to the left breast/chest wall was considered as high-risk RT and was associated with an increased risk of coronary artery stenoses in mdLAD + dD, with a 4- to 7‑fold risk increase in significant stenoses for the mid and distal LAD in radiation hotspot areas. The authors concluded that the findings indicate a direct link between radiation and the location of coronary stenosis.

Moignier et al. analyzed the risk of coronary stenosis following Hodgkin lymphoma RT of the mediastinum [23]. The authors performed a 3D coronary artery dose calculation after mediastinal RT using coronary CT angiographies. Twelve patients developing coronary stenosis after mediastinal RT were matched to 21 irradiated patients without stenosis. Radiation doses to stenotic segments were compared with doses to normal segments. Based on these findings, the authors estimated the risk of coronary stenosis depending on the radiation dose to the coronary arteries. It could be shown that the coronary artery segment dose significantly increased the risk of stenosis in the segment. The median dose to the damaged vs. undamaged coronary segments was 30.3 vs. 26.3 Gy (25^th^ to 75^th^ percentile: around 26 to 40 Gy vs. 3.3 to 35 Gy; *p* < 0.001).

### Functional imaging

Marks et al. analyzed myocardial perfusion in the cardiac apex 6, 12, 18, and 24 months after RT to the left breast [24]. In this prospective trial, 114 patients with left-sided breast cancer were treated with 46–50 Gy using tangential photon beams. By inclusion of the cardiac apex into the radiation fields (by the authors’ definition, equivalent to >50% of the prescribed dose), new perfusion defects were detected in 27, 29, 38, and 42% of patients after 6, 12, 18, and 24 months, respectively. If <5% versus ≥5% of the left ventricle was included into the radiation portals, perfusion defects were seen in 10–20% vs. 50–60% of patients, respectively.

### Strain rate imaging (Doppler echocardiography)

Erven et al. analyzed early radiation-induced changes in regional cardiac function using the strain-rate imaging (SRI) method by Doppler echocardiography [25]. The authors included 20 left-sided and 10 right-sided breast cancer patients irradiated to the breast or chest wall. Echocardiography and SRI were performed before and immediately after the RT course and repeated 2 months thereafter. The LV was divided into 18 segments. Regional strain and strain-rate values were analyzed from all segments and related to the radiation dose applied to the corresponding region. For the left-sided patients, a strain and strain-rate reduction could be seen post RT. In the apical segments receiving >3 Gy vs. <3 Gy, a significant decrease in strain expressed as a decrease in systolic myocardial deformation could be observed.

In line with this data, Heggemann et al. saw a decrease in longitudinal strain in apical segments after 24 months [26]. In this study, recorded doses to the apex were 34.4 ± 10 Gy.

### Serum biomarkers

Focusing on cardiac biomarkers, D’Errico et al. found indications that not the mean dose but the percentage of organ volumes receiving doses much higher than the mean heart dose are relevant [27]. The authors demonstrated that cardiac biomarkers such as N‑terminal pro-B-type natriuretic peptide (NT-proBNP) or troponin (TnI) increase after left-sided breast RT and evaluated the correlation between the respective plasma levels and the radiation dose to the heart. At mean 9 months after left-sided RT of the breast, NT-proBNP levels were significantly higher compared to those of non-irradiated patients. No significant correlation was noted for mean heart dose and the biomarker levels. In contrast, the dosimetric parameters V3_heart_ (percentage volume of the whole heart receiving ≥ 3 Gy) and V2_LV_ (percentage volume of the LV receiving ≥2 Gy) correlated significantly with NT-proBNP. Furthermore, several other dosimetric parameters were analyzed, focusing on small heart volumes receiving higher doses. For example, the increase of the ratio $\text{D}_{15{\text{cm}^{3}}}$ (Gy)/D_mean_ (Gy) correlated significantly with the level of NT-proBNP. The authors stated that the most important parameter is not the mean dose, but rather the percentage of organ volumes receiving doses much higher than the mean heart dose.

Skyttä et al. found a positive correlation between cardiac doses and the serum biomarker troponin T (high-sensitivity cardiac troponin T, hscTnT) [28]. In a prospective study, hscTnT was analyzed before, during, and immediately after finishing the course of RT. An increase in hscTnT of > 30% was interpreted as significant. In patients with such an hscTnT increase, the mean heart dose and mean LV dose were significantly higher (4 vs. 2.8 Gy, *p* = 0.02 and 6.7 vs. 4.5 Gy; *p* = 0.02). Furthermore, the mean LAD dose (17.5 vs. 23.8 Gy) and V15 (58.6 vs. 40%), V20 (55.4 vs. 36.2%), and V30 (45 vs. 29.3 Gy) for the LAD volume were significantly higher. The maximum LAD dose was also higher (43.4 vs. 37.8 Gy), but not significantly.

A summary of several findings focusing on radiation-induced heart toxicity (see above) and deduced dose constraints for heart and subvolumes is given in Table 2.

## Discussion

Even small heart doses are suspected to increase the risk of cardiac disease. Darby et al. estimated the proportional increase in the rate of major coronary events per Gray. This assumption was based on a retrospective evaluation using a population-based case–control study. Darby et al. found that the risk of major coronary events (i. e., myocardial infarction, coronary revascularization, deaths from ischemic heart disease) increased linearly with the increase in mean heart dose with no clear threshold [29]. They showed a dose–effect relationship with an increase in the relative risk of acute major coronary events of 7.4% per Gy (95% confidence interval, CI: 2.9–14.5; *p* < 0.001) mean heart dose within 20 years [29]. The increase started within the first 5 years after RT and continued into the third decade after RT. Although women with preexisting cardiac risk factors had greater absolute increases in the risk from RT, the proportional increase in the rate of major coronary events per Gy was similar in women with and without cardiac risk factors at the time of RT. Of note, for mean heart doses below 2 Gy, no significantly increased event rates were seen.

Recently, the findings of Darby et al. were validated by van den Bogaard et al. [30]. The authors analyzed 3D dose distributions to the heart and cardiac substructures derived from CT planning scans of an independent cohort of patients with breast cancer treated by RT. Validating the model of Darby et al., the authors created a multivariable Cox regression model using the same prognostic and pretreatment risk factors (i. e., age, mean heart dose, history of ischemic heart disease, diabetes, chronic obstructive pulmonary disease, smoking, body mass index ≥30 kg/m^2^).

Van den Bogaard et al. found a relative increase in the cumulative incidence of acute coronary events (ACE) of 16.5% per Gy (hazard ratio, HR: 1.165; 95% CI for HR: 1.006 to 1.350; *p* = 0.042) of mean heart dose within 9 years of RT.

Also for other tumor entities including lung cancer, Hodgkin lymphomas, or after mediastinal irradiation, the mean heart dose is known to be a relevant parameter for prediction of all-cause cardiac toxicities [31].

Examining the context of coronary heart disease and RT, interesting data were yielded by Carr et al. based on a patient cohort treated with RT for peptic ulcer disease [32]. Although this treatment indication is historical, the data provide indications to further understand the relationship between total heart dose and the dose to the apex. The authors analyzed data of 3719 patients irradiated for peptic ulcer disease between 1937 and 1965 using orthovoltage X‑rays encompassing the stomach by anterior and posterior opposing fields. The daily and total doses were 1.5 and 16–17 Gy, respectively. The authors estimated that 5% of the cardiac volume, generally apex volume, was included into the radiation field and received 7.6–18.4 Gy. The estimated dose to the cardiac volume outside of the radiation field was 1.6–3.9 Gy.

The authors found a statistically significant increase in coronary heart disease in patients with an estimated whole heart dose of 2.8 Gy and 12.9 Gy to 5% of the cardiac volume (relative risk 1.54; 95% CI: 1.15–2.06). A mean whole heart dose of 1.6 Gy accompanied with an in-field dose (to the apex) of 7.6 Gy led to no increase in the relative risk for coronary heart disease.

### Time-factor

For a comprehensive assessment of coronary artery disease (CAD) risk in the context of breast irradiation, the time factor seems to be highly important, but data related to this are not entirely consistent.

Darby et al. impressively demonstrated that the risk of CAD continuously increased with time after finishing breast irradiation. The increase in risk began within the first 5 years after radiation and continued for at least 20 years [7]. Considering all major coronary events detected within the time span of 0 to 20 years after RT, the relative risk increased by 7.4% per Gy. Interestingly, in the study by Darby et al., the strongest increase in relative risk of major coronary events was seen within the first 4 years, with a rate of 16.3% per Gy.

To examine whether the risk of cardiac death was higher in the second than in the first follow-up decade after RT, Harris et al. performed a cumulative hazard risk estimation based on data of patients irradiated between 1977 and 1994 [9]. For left-sided patients, the cumulative risk of cardiac deaths was 1.9% (95% CI, 0.09 to 3.9%) after 10 years and 6.4% (95% CI, 3.5 to 11.5%) after 20 years. In comparison, for right-sided radiation, the cumulative risk increased from 1.5% to 3.6% in the same time span.

## Further factors affecting cardiac risk

It should be acknowledged that several other factors affect the cardiac risk. The risk for cardiotoxicity as well as its severity depends on many factors and is further determined by the presence of traditional cardiovascular risk factors, particularly cardiometabolic risk factors such as diabetes mellitus, arterial hypertension, dyslipidemia, and obesity. Preexisting cardiovascular diseases such as arrhythmia, myopathy, or chronic ischemic heart disease represent further risk factors.

### Smoking

Smoking is a highly relevant risk factor potentiating the risk of radiogenic heart damage after left-sided breast RT. The increase in absolute risk in radiation-related cardiac mortality is more pronounced in smokers than in nonsmokers. In general, the mortality from heart disease is much higher for smokers than nonsmokers. Based on European female death rate data, the estimated risk of death before reaching an age of 80 years was 1.8% for a nonsmoker and 8.0% for a smoker [33]. Based on these data and supposing a mean heart dose of 4.4 Gy, Taylor et al. calculated an absolute increase in cardiac mortality related to RT of 0.3% for nonsmokers (1.8 to 2.1%) and 1.2% for smokers (8.0 to 9.2%) [34].

### Systemic treatments

The contribution of chemotherapy in addition to RT remains an important aspect in the development of cardiac disease in cancer patients and plays an important role as a further risk factor. Anthracyclines and trastuzumab are notorious anticancer drugs and responsible for the development of cardiac disease.

Chemotherapy-induced cardiotoxicity is distinguished into type 1 (anthracycline) and type 2 (trastuzumab). In type 1, structural damage to the cardiomyocytes is induced and must be considered irreversible. Type 2 is characterized by the lack of structural changes, so that the end of therapy usually brings complete recovery; thus, the damage is reversible. Other substances with cardiotoxic effects are, for example, cyclophosphamide, clofarabine, fluorouracil, vincristine, interferon-alpha-2b, sunitinib, and sorafenib [35, 36].

### Risk-factor assessment

To improve the safety of patients with breast cancer before starting chest RT, patients should undergo a baseline assessment for RIHD risk factors and, in case of preexistent risk factors, a thorough clinical examination, a baseline echocardiography evaluation, and possibly further diagnostics as recommended by the European Association of Cardiovascular Imaging of the European Society of Cardiology and the American Society of Echocardiography expert group [37–39].

## Value of mean heart dose

The key question is: Is the mean heart dose able to predict the risk of acute cardiac events?

In principle, mean heart dose seems to be a valid parameter for predicting cardiac toxicity. It is well-documented that reducing the mean heart dose is associated with lower risks of cardiac late effects [10, 12, 22, 29, 40]. Using modern techniques for breast irradiation, low mean heart does in a range below 2–3 Gy are achievable. Despite such low mean heart doses, subvolumes such as the heart apex or parts of the LAD can be exposed to much higher doses (Figs. 1 and 2; [41]). In a study conducted by the authors using a modern 3D technique with tangential beams to treat left-sided breast cancer, the mean heart dose amounted 2.1 (0.98–8.3) Gy [42]. Nonetheless, maximum doses to small but presumably relevant parts of the anterior part of the LV (“anterior myocardial territory,” AMT; based on Tan et al. [43]) were up to 47.2 Gy. The mean and maximum doses to the LAD were 9.2 (2.1–46.2) Gy and 24.6 (2.8–49.6) Gy, respectively.

The problem is illustrated in Figs. 1 and 2. In Fig. 1, radiation treatment planning scans (transversal planes) of the left breast are shown. In both cases, modern 3D planning was performed. Even without any specific heart-protecting technique, the mean heart dose is below 2.5 Gy. Nevertheless, apical areas like the LAD and LV receive much higher doses.

The question of which parts of the heart are of highest relevance for late effects—thus implying the necessity of their optimal protection—is not definitively answered. Without prejudice to the fact that there is an increased radiogenic risk for cardiac damage after left-sided breast cancer RT, there are no clear constraints and thresholds with respect to absolute doses and (sub)volumes.

Considering the available data, it seems to be reasonable to define several parts of the heart, especially the anterior part, as organs at risk (OAR). For example, in a comparative dosimetric study, Tan et al. demonstrated that using the AMT as an OAR in left-sided breast intensity-modulated RT (IMRT), the radiation dose to the heart could be reduced [43, 44].

But what is the threshold dose for heart disease regarding the whole heart and its substructures? What is a potentially safe dose cut-off?

To date, based on the available literature and considering the lack of more detailed prospective data, the dose constraints to heart and subvolumes shown in Table 3 seem to be reasonable.

**Table 3:** Dose constraints for heart and substructures in breast radiotherapy

Volume	Constraint	Value
Whole heart	Mean heart dose	<2.5 Gy
Left ventricle	D_mean_ LV	<3 Gy
	V5_LV_	<17%
	V23_LV_	<5%
LAD	D_mean_LAD	<10 Gy
	V30_LAD_	<2%
	V40_LAD_	<1%

*LV* left ventricle; *LAD* left anterior descending artery; *D*_*mean*_ mean dose of the volume; *Vx*_*LV*_ percent of left ventricle volume receiving ≥ x Gy

More restrictively, considering the data from Carr et al. [32] for the whole heart, a mean heart dose <1.6 Gy and a V13 < 5% could also be justifiable. As a matter of course, doses to the heart and subvolumes should be kept as low as possible.

## Radiation to the breast and regional lymph nodes

The suggested constraints have largely been developed for adjuvant whole-breast RT. In cases of comprehensive regional irradiation including the internal mammary lymph nodes, exceeding these constraints may be unavoidable and justifiable.

## Hypofractionation

Hypofractionation (40–42.5 Gy with daily doses of 2.5–2.67 Gy) has recently become the standard for adjuvant RT to the breast [45]. The available data for estimation of radiation-induced cardiac risks mostly refer to normal fractionation regimes, but are these experiences applicable to hypofractionation regimes?

From a radiobiological perspective, heart and coronary vessels are late-responding tissues. Generally, an α/β value = 3 Gy has been assumed for late-responding tissues and based on rat heart studies, even lower values may be suggested, possibly as low as 1 Gy [46, 47]. Such tissues are particularly sensitive to increasing fraction doses. Appelt et al. estimated the fraction size-corrected dose to the heart for hypofractionation regimens based on the linear quadratic model [47]. Dose distributions of hypofractionated treatment plans were corrected to the equivalent dose in 2 Gy fractions (EQD2) using the linear quadratic model for normal fractionation and four hypofractionation regimens. The tested range of α/β values was from 0 to 5 Gy. The authors stated that for α/β ≥ 1.5 Gy, the hypofractionation regimens using 40 Gy (2.67 Gy daily), 42.5 Gy (2.65 Gy daily), and 39 Gy (3 Gy daily) result in lower equivalent doses to the heart than the normal fractionation regime (50 Gy/2 Gy).

These findings are in line with the clinical results of the randomized Canadian and Start B trials, where no increased cardiac toxicity was seen in the hypofractionation treatment arms [48, 49]. Recently, James et al. also observed no differences in ischemic heart toxicities when comparing normal and hypofractionated breast treatment [50].

## Technical options reducing heart dose

**Fig. 3:**
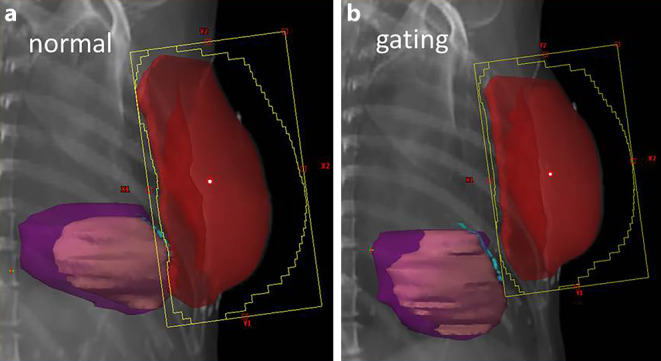
Digital reconstructions from the two treatment plans. **a** Planning with normal breathing without deep-inspiration breathold (DIBH); **b **the same patient planned using gating with DIBH. Planning target volume in *red*, heart in *purple*, left ventricle in *bright purple*, left anterior descending artery contoured in *light blue*

Several technical options are available to limit the mean heart dose or to specifically spare selected subvolumes of the heart such as the coronary arteries. Whereas tangential IMRT or field-in-field approaches are useful to avoid hotspots at the skin or within the breast in order to improve cosmetic outcome and reduce the risk of fibrosis, no relevant sparing of the heart and lungs can be achieved [51]. Multiangle or rotational IMRT delivery can be used to create concave dose distributions and to reduce the high dose volume of the lung and heart abutting the chest wall at the cost of a low-dose bath to the ipsi- and contralateral lung and the whole heart [52]. DIBH-based radiation therapy can help to distance the heart from the chest wall and reduce the dose to the heart and substructures such as the LAD [53–56]; Figs. 1, 2 and 3. In selected cases, especially with pendulous breasts, prone positioning can result in favorable geometry with distancing of the target volume away from the chest wall, whilst at the same time moving the heart closer to the chest wall. Alternatively, the usefulness of a thermoplastic bra in terms of dose reduction to heart and lung substructures has been demonstrated [42]. Finally, partial-breast RT is an option in elderly patients with low-risk cancer, especially when no adequate sparing of heart and lung can be achieved during WBI [57–59].

## Conclusion

Heart toxicities due to RT of the breast—particularly left-sided breast RT—are rare but clearly recognizable. Modern techniques permit sufficient protection of the heart and lungs in most cases. However, in some instances, i. e. in patients with unfavorable anatomy, subvolumes of the heart—particularly apical regions such as the LV or the LAD—receive high doses despite low mean heart doses.

Valid data defining dose constraints to subvolumes of the heart are sparse. In the current report, the authors propose dose constraints to the heart and its subvolumes to achieve an adequate heart protection and which may be achievable in conventional and hypofractionated regimens. The suggested constraints apply to left-sided breast RT only. For several kinds of breast irradiation, particularly if lymph nodes must be included, these constraints are not achievable.

Furthermore, patient-specific cardiac risk factors and the individual breast cancer-related risk constellation must be considered. The patient’s breast cancer mortality risk and cardiac risk factors must be individually interrelated to possible radiation-induced heart toxicities. The final and individual decision between protection of heart volumes and target volume coverage remains in the physician’s hand.
